# Microalgae Harvesting Using Ceramic Membranes: Semi-Industrial Scale Study

**DOI:** 10.3390/membranes16040132

**Published:** 2026-04-01

**Authors:** Stacy Ragueneau, Clémence Cordier, Adeline Lange, Laurent Torres, Philippe Moulin

**Affiliations:** 1 Aix Marseille Univ. CNRS, Centrale Med., M2P2 UMR 7340, Equipe Procédés Membranaires (EPM), Europôle de l’Arbois, BP80, Pavillon Laennec, Hall C, 13545 Aix en Provence Cedex, France; rgn.stacy@gmail.com (S.R.); clemence.cordier@univ-amu.fr (C.C.); 2 Innovalg, Polder des Champs, 85230 Bouin, France; afl25a@fsu.edu (A.L.); laurent.torres@innovalg.fr (L.T.)

**Keywords:** microfiltration, microalgae harvesting, ceramic membranes

## Abstract

Microalgae, being able to produce a variety of bioactive compounds, represent a promising resource for numerous industrial applications. However, their large-scale production remains constrained by biological, technical and economic factors. Open ponds, which are predominantly employed on an industrial scale, yield lower levels of algae in comparison to those obtained in closed reactors. Consequently, the processing of substantial volumes is necessitated during the harvesting process. This study explores the potential of microfiltration as an alternative to conventional harvesting processes to optimise yields and preserve biomass quality. The evaluation of various ceramic membranes, including new-generation prototypes, was conducted according to several operating parameters (flux, backwash mode, recirculation rate). The objective was to obtain microalgae concentrate while preserving cell integrity. Three species (*Odontella aurita*, *Phaeodactylum tricornutum* and *Dunaliella salina*) were considered for issues directly related to industrial cultivation such as seasonality, strain variability and the state of the culture at the time of harvest. An effective cleaning protocol was also developed, applicable to all the conditions tested. The ceramic membranes demonstrated a high degree of resistance to fouling, with their low tortuosity promoting effective backwashing. The membrane process resulted in a high level of cell recovery and volume concentration factors that were comparable to those achieved by conventional methods. In comparison with alternative concentration processes, it is also economically viable, thus confirming its potential as a robust and efficient alternative for industrial-scale microalgae harvesting.

## 1. Introduction

The utilisation of microalgae is gaining increased interest due to their potential in various domains, including food, cosmetics, bioenergy and green chemistry. These photosynthetic microorganisms have been found to be a rich source of proteins, lipids, pigments and bioactive compounds, while also contributing to CO_2_ biofixation and water purification [1,2,3]. However, the industrial production of microalgal biomass is constrained by the harvesting stage, which can account for 20 to 30% of total costs [4,5]. The objective of this stage is to separate the biomass from the culture medium, with a view to facilitating downstream processes such as the extraction of bioactive compounds. The cells exhibit a small size (1–30 μm), low density and dispersion in the culture medium, which collectively render them challenging to separate efficiently [6]. A number of processes are utilised for the collection of biomass, including filtration, centrifugation, flocculation and flotation [7]. In certain instances, a combination of multiple processes is favoured to enhance the efficiency of the harvesting process. Centrifugation is a widely used method due to its efficiency (>90% recovery) and low maintenance requirements. However, this process remains very energy-intensive (up to 8 kWh m^−3^) and can damage cells due to the centrifugal force [8,9,10]. While it remains suitable for high-value-added production, its large-scale application is limited by these constraints [11]. Flocculation is an intriguing alternative, as it results in the aggregation of cells into larger structures. Chemical flocculants, such as aluminium and iron salts, offer high recovery efficiency (approximately 95%), but pose toxicity issues and require additional purification steps [12,13]. Bioflocculants, such as chitosan, appear to be a more sustainable option [14,15], although their cost and variable effectiveness depending on the species limit their adoption. An alternative approach is predicated on flotation, which exploits the adhesion of microbubbles of air to cells, thereby facilitating their separation [7,16,17,18,19]. Despite their effectiveness, these processes remain limited by energy consumption and maintenance issues [20]. Finally, membrane filtration is emerging as a promising process thanks to its near-total cell retention and optimised energy consumption if fouling is controlled [7,9]. The selection of membrane (Microfiltration: MF, Ultrafiltration: UF), the composition of its material (organic or inorganic), and its configuration (hollow fibre, tubular, spiral) exert a substantial influence on the performance of the process [21,22,23]. Given that the size of microalgae cells generally ranges from 1 to 30 μm, MF membranes (0.02 to 10 μm) and UF membranes (0.001 to 0.01 μm) are most commonly used for harvesting them [9]. A number of studies have indicated that membranes with larger pores offer enhanced performance [21,23], while other research has yielded contradictory results, suggesting that MF membranes tend to be more sensitive to fouling and then experience a more pronounced decline in permeability than UF membranes [22,24,25,26]. One hypothesis suggests that microalgae cells become embedded in the pores of MF membranes, resulting in irreversible fouling. Conversely, with UF membranes, microalgae cells settle on the surface, leading to reversible fouling. However, this is contingent on the characteristics of the filtered microalgae suspension. The impact of pore size distribution on filtration performance is not the only factor to be considered; the surface properties of the membrane and the underlying porosity must also be taken into account, as must the concentration of extracellular organic matter and the density, size and shape distribution of microalgae, as these factors influence the viscosity, flux and mixing of solutions [27,28]. The utilisation of organic membranes, such as polyvinylidene fluoride (PVDF), is prevalent due to their cost-effectiveness [29]. However, their vulnerability to high pH levels and temperatures during the washing stages limits their longevity [30,31]. Inorganic membranes, including ceramic membranes (TiO_2_, α-Al_2_O_3_), demonstrate superior resistance and recovery efficiency, with values reaching up to 98%. However, their cost remains a significant obstacle [32,33,34]. Despite being the subject of less research than organic membranes, inorganic membranes have demonstrated their efficacy in the harvesting of various microalgae species, including *Arthrospira platensis*, *Scenedesmus obliquus*, *Tisochrysis lutea*, *Porphyridium cruentum*, *Nannochloropsis salina*, *Nannochloropsis limnetica*, *Chlorella* sp. and *Desmodesmus* sp. [32,33,34,35]. As demonstrated in the works of Malaguti et al. (2024), Ricceri et al. (2022) and Safafar (2017) [32,33,35], the pertinent literature on the subject is as follows: At the laboratory scale, ceramic membranes have been shown to achieve recovery rates of up to 98% [33,35]. However, the efficiency of the process varies depending on the species studied: *Phaeodactylum tricornutum* exhibited the optimal concentration ratio, while *Monodopsis subterranea* demonstrated substandard performance. Consequently, despite the numerous advances witnessed in the field, no harvesting method has yet emerged as a universal solution. Each approach involves a trade-off between energy efficiency, cost, environmental impact and preservation of cell integrity. Whilst centrifugation and flocculation have long been considered reliable and established processes, membrane and flotation technologies are beginning to emerge as promising alternatives [36,37]. However, it is essential to note that the viability of these alternatives is subject to the optimisation of fouling in the case of membrane process, the reduction in energy consumption, and the limitation of chemical contamination. The selection of the process is thus contingent upon the microalgal species in question, the culture conditions, the concentration objectives and the anticipated purity of the biomass. In this context, the scientific and industrial challenge is to develop hybrid and integrated processes, whatever the microalgae, that reconcile yield, sustainability and economic feasibility for competitive large-scale microalgae production [34,35]. The present study will focus on the semi-industrial development of microfiltration using ceramic membranes, particularly silicon carbide (SiC) membranes, for the preconcentration of microalgae cultures with a view to coupling with a centrifugation step. The objective was to investigate and optimise SiC microfiltration for the purpose of microalgae harvesting in an industrial context. In addition to considerations of species diversity, potentially highly variable parameters such as culture concentration and purity must also be considered, given their significant impact on membrane fouling in industrial production. The study’s primary objective was to analyse the performance of the process, the efficiency of cleaning, the rates of retention and concentration factors, and the associated costs.

## 2. Materials and Methods

### 2.1. Biological Material and Measurements

The microalgal species examined in this study are *Odontella aurita*, *Phaeodactylum tricornutum*, and *Dunaliella salina*. The *P. tricornutum* strain has been isolated from seawater collected in Bourgneuf Bay (Vendée, France), which also supplies the Innovalg production site. Similarly, *D. salina* was isolated in 2022 during high-salinity culture tests (>300 g L^−1^) [38]. In the case of *O. aurita*, it was initially isolated from seawater, and the strain was later renewed through the acquisition of external strains, notably AC815 and AC816 from Algobank (Caen, France). All three species are cultivated by Innovalg in outdoor open ponds agitated by paddle wheels. The algal cultures employed in this study were obtained from production ponds (100 m^3^), except for *D. salina*, which was cultivated in a 300 L indoor cylindrical reactor. Each species is produced at different times of the year to avoid cross-contamination, with no overlap in outdoor cultivation periods. The cultures are grown in a mixture of natural seawater and iron-rich borehole seawater. Outdoor systems are supplied with CO_2_ to maintain a pH of 8.5, while indoor cultures receive air enriched with 1% CO_2_. Nutrient supplementation differs between systems: outdoor cultures are supplied with a concentrated nutrient solution (500 g L^−1^ NaNO_3_ and 94 g L^−1^ H_3_PO_4_), whereas indoor cultures are enriched using Conway or Walne medium [39]. Microalgal cell concentrations were determined by direct counting using a hemocytometer and optical microscopy at 40× magnification. Dry weight measurements were obtained by filtering 100 mL of algal suspension through pre-weighed 47 mm Whatman GF/B filters (1 μm pore size) (Whatman, Maidstone, UK). The filters were rinsed with ammonium formate (35 g L^−1^, 540-69-2, Dutscher, Bernolsheim, France), dried at 90 °C for 4 h, and subsequently reweighed to determine dry mass.

**Figure 1:**
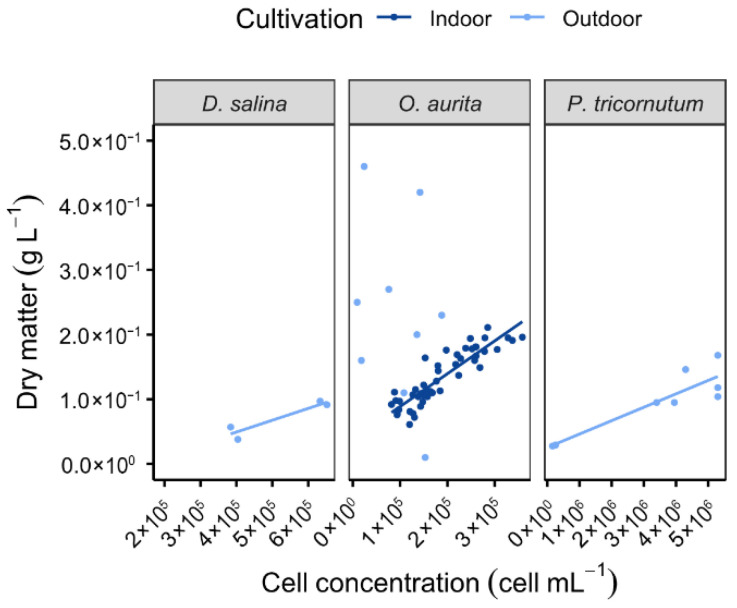
Variation in the dry matter as a function of initial cell concentration of the harvested cultures according to the species studied and to indoor or outdoor cultivation mode [40].

In outdoor production systems, parameters such as pond concentration, contamination level, and culture cleanliness exhibit significant variability, directly influencing the performance of membrane filtration. *O. aurita* cultures are frequently impacted by recurrent contamination with ciliates, rotifers, and other microalgae, including *D. salina* and *P. tricornutum*. The impact of these contaminants is illustrated in Figure 1, which shows the relationship between dry matter content and cell concentration in the suspensions of the three species, measured immediately before harvesting. For *D. salina* and *P. tricornutum*, a clear proportional relationship was observed between cell concentration and dry matter for outdoor conditions. In contrast, this correlation was not evident for *O. aurita*, likely due to the presence of additional organic and inorganic compounds in the culture. *O. aurita* appears to demonstrate a degree of tolerance when co-cultivated with contaminants: the presence of other microalgae does not inhibit its growth, but it cannot outcompete them. Furthermore, the organism exhibits limited vulnerability to predators, likely due to its relatively large cell size. Consequently, *O. aurita* is harvested irrespective of its culture state, although the biomass may occasionally contain other microalgae or small grazers. The influence of contamination is further highlighted through a comparison of dry matter concentrations between outdoor and controlled indoor cultures, where the proportional relationship between cell concentration and dry matter is evident.

All experiments conducted as part of this study were carried out using industrial cultures sourced from Innovalg’s production basins. Given the semi-industrial scale of the study, each test was carried out using a single culture with unique characteristics in terms of composition, cell concentration and dry matter concentration. This ensured that the diversity and variability inherent in industrial-scale production were represented. Due to the substantial quantity of tests conducted, it proved unfeasible to carry out a complete characterisation of the cultures utilised on an individual basis. The parameters that were measured were cell concentration and dry matter; however, the number and nature of potential contaminants could not be determined.

### 2.2. Microalgae Pre-Concentration Methods

#### 2.2.1. Microfiltration Unit and Membranes

The filtration parameters of the membrane process were optimised using a semi-industrial microfiltration unit [41]. The unit is capable of operating with one or two membranes, which are arranged in a parallel configuration. The system incorporates a production circuit and an independent washing circuit, enabling semi-autonomous operation during the production phase. This process is facilitated by automated regulation of operating parameters, including permeate, concentrate, and recirculation flux rates, in addition to the triggering of backwashing. The process variable parameters (i.e., flux rates, pressures, temperatures and tank levels) are recorded at a rate of three seconds. During the course of the production process, the suspension to be treated was introduced into a 200 L feed tank that was equipped with a propeller agitator (PBC 1.18-4007-1-130, Inoxpa, Girona, Spain; 1420 rpm). This agitator ensured constant homogenisation, thereby maintaining the particles in suspension. The selection of centrifugal pumps was driven by the necessity to facilitate the filtration of shear-sensitive microalgae. In circumstances where elevated levels of heat were observed as a consequence of recirculation, the suspension was subjected to cooling through the utilisation of a heat exchanger. The pilot plant was equipped with a feed pump and a recirculation pump. This recirculation loop has been demonstrated to limit heating; however, as demonstrated by Monte et al. (2020) [42]. However, it has also been shown to impose limitations on the pressure compression phases of an open-loop pilot plant, which can have a detrimental effect on cell integrity. The permeate was collected in a dedicated 70 L tank, which was also used for backwashing. The concentrate was retained within the filtration loop, with the exception of periods during backwashing phases, when the water from backwashing was returned to the feed tank, carrying the accumulated concentrate with it. The rationale behind the decision to refrain from draining the concentrate was to optimise the concentration factors. Consequently, the concentrate was reintroduced to the feed tank during each backwash cycle, thereby enhancing the concentration of the feed suspension as the filtration process unfolded. Irrespective of the suspension concentration, the permeate flux diminished due to fouling, which escalated with the concentration upstream of the membrane, even in tangential filtration. In order to concentrate the initial suspension to a high degree, it was essential to perform backwashing, which resulted in a reduction in transmembrane pressure (TMP) at constant flux. This, in turn, increased the permeability of the membrane. The unit performed two types of backwashing: (i) backwashing (BW) whereby the permeate is injected in countercurrent during the pressure increase from 0 to 2 bar and then for 5 s, and (ii) backpulsing (BP), where the target pressure (3 bar) was reached before pressurised permeate was injected into the membrane in countercurrent for 5 s, increasing the contact force with the membrane while reducing the volume of water reinjected into the system. In the context of chemical cleaning procedures, the membranes were meticulously maintained through systematic cleaning protocols that were implemented post-filtration cycle. The operation was initiated with a freshwater rinse, followed by backwashing to remove as much surface fouling from the membranes as possible. The chemical cleaning itself was carried out in a closed loop in a 70 L tank equipped with a heating element to minimise reagent consumption. The procedure entails a preliminary hot wash, comprising NaOH at a concentration of 30 g L^−1^ at an approximate temperature of 60 °C for a duration of 1 h. This was followed by a rinsing step, and subsequently, an acid wash was conducted using HNO_3_ at a concentration of 0.25% for a duration of 30 min. As part of the semi-industrial scale tests, a tolerance threshold of ±15% around the initial reference permeability was established to assess the effectiveness of the cleaning process. The membranes utilised in this process were LiqTech (Ballerup, Denmark) (Manufacturer A) mineral microfiltration multi-channel tubular membranes (Table 1). The LiqTech SiC exhibits a molecular weight cut-off of 0.2 µm, 30 channels with a diameter of 3 mm, a filtration area of 0.33 m^2^ and a water permeability of 7000 L h^−1^ m^−2^ bar^−1^. A comparative analysis was conducted with another SiC membrane (Manufacturer B), possessing a molecular weight cut-off of 0.25 µm, 31 channels with a diameter of 3 mm, and the same geometry and filtration area, but with water permeability of 13,500 L h^−1^ m^−2^ bar^−1^. The selection of silicon carbide as the material was informed by its notably high water permeability, its notable resistance to pH levels ranging from 0 to 14, and the low tortuosity of the material (~1.2) [43] in comparison with ceramic membranes. It should be noted that each test presented in this paper was performed in duplicate because, for each manufacturer, two identical membranes, M1 and M2, were used in parallel on the filtration pilot. Thus, in the rest of the text, Manufacturer A and Manufacturer B refer to the two types of membranes compared from two manufacturers (Table 1), and M1 and M2 refer to the duplicates performed for each filtration test regardless of the manufacturer.

The analysis of filtration performance involved the evaluation of various fouling resistances utilising the series resistance model (Equation (1)). The total resistance R_T_ is equivalent to the sum of all resistances acting on the membrane. The membrane resistance R_m_ is defined as the specific contribution of the membrane material and is indicative of its resistance to mass transfer.

(1) $$Lp=\frac{1}{µ\times R_{T}}=\frac{1}{µ\times (R_{m}+R_{irr}+R_{rev})}$$

where R is the resistance to mass transfer in m^−1^, Lp the permeability of the membrane in m^3^ s^−1^ m^−2^ Pa^−1^ and µ is the dynamic viscosity of the fluid in Pa s.

Reversible resistance (R_rev_) was calculated using the following equation:

(2) $$R_{rev}=R_{T\;end\;of\;cycle\;n}-R_{T\;start\;of\;cycle\;n+1}$$

where R_T end of cycle n_ is the total resistance at the end of filtration cycle n, in m^−1^, and R_T start of cycle n+1_ is the total resistance at the start of the next cycle.

Irreversible resistance (R_irr_) was calculated using the following equation:

(3) $$R_{irr}=R_{T\;start\;of\;cycle\;n}-R_{m}$$

where R_T_ is the total resistance at the start of cycle n in m^−1^, and R_m_ is the resistance of the membrane.

Finally, shear stress (τ) in Pa was calculated using the following equation:

(4) τ=µ×γ

with γ the shear ratio in s^−1^ and μ the dynamic viscosity in Pa s. The dynamic viscosities of the microalgae cultures were estimated using a rheometer and are in the range between 0.00107 Pa s and 0.00118 Pa s for seawater and a concentration of 800,000 cells mL^−1^ of *O. aurita*.

#### 2.2.2. Flocculation and Decantation

The concentration results obtained by membrane filtration were compared with the conventional process performance achieved by flocculation and/or decantation, depending on the species of algae studied. For *P. tricornutum* and *D. salina*, the culture was transferred from the outdoor tanks to two settling tanks (2.6 m^3^). The administration of the flocculant was initiated immediately upon the initiation of the culture within the tank. Once the tank was full, a contact time was observed, after which the supernatant was automatically drained. The process was then repeated with a maximum of 12 cycles, with the tanks being replenished. Following the conclusion of the designated cycles, the concentrate that had accumulated at the base of the tanks was transferred to a single tank. This step enabled a final decanting phase and, if necessary, pH neutralisation at 7.5. Indeed, the flocculant employed varied according to the species in question. For instance, in the case of *P. tricornutum*, sodium hydroxide (0.063 g L^−1^) was utilised initially, followed by acid neutralisation with hydrochloric acid (0.015 g L^−1^). Conversely, in the case of *D. salina*, ferric chloride (0.003 g L^−1^ FeCl_3_) was employed. In the case of soda, the pH was adjusted to 7.5 prior to the continuation of the process. Finally, *O. aurita* is a large microalga with low-concentration cultures, so its harvest involves a pre-concentration step using a 15 µm drum filter. A salient feature of this species is its capacity for natural sedimentation, thereby eliminating the requirement for the incorporation of flocculants. The concentrate obtained was then transferred to settling tanks, and the settling and centrifugation phases were carried out in a similar way to other species, but without the use of chemicals.

## 3. Results and Discussion

### 3.1. Membrane Selection

**Figure 2:**
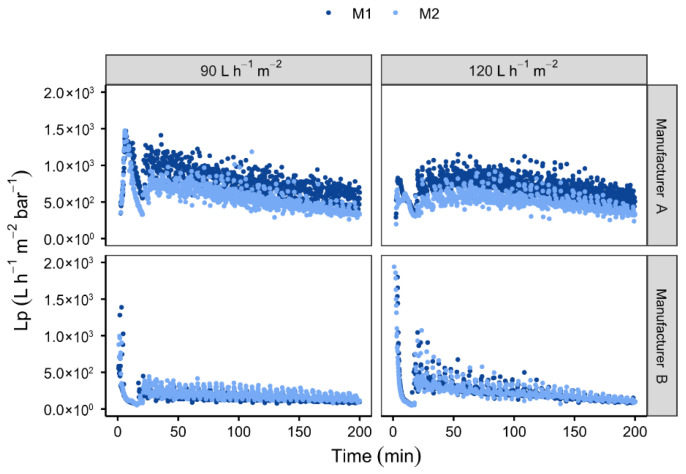
Variation in membrane permeability as a function of time for two membranes from different manufacturers (A and B) and for two permeate fluxes: 90 and 120 L h^−1^ m^−2^. [Backwash type: BP; BP frequency: 10 min; Recirculation: 3500 L h^−1^; Microalgae: *O. aurita*].

The membranes under consideration were selected following a preliminary testing process, not detailed in this paper [44], which consisted of comparing the fouling resistance of membranes from two manufacturers with similar characteristics. The initial SiC membrane, manufactured by LiqTech (Manufacturer A), exhibits a molecular weight cut-off of 0.2 µm, 30 channels with a diameter of 3 mm, a filtration area of 0.33 m^2^, and a water permeability of 7000 L h^−1^ m^−2^ bar^−1^. A comparative analysis was conducted with a SiC membrane manufactured by a different company (Manufacturer B). This membrane possesses a molecular weight cut-off of 0.25 µm, 31 channels with a diameter of 3 mm, and the same geometry and filtration area. However, it demonstrates superior water permeability of 13,500 L h^−1^ m^−2^ bar^−1^. The experimental procedure involved the execution of tests on both membranes, with two identical membranes (from the same manufacturer) utilised in parallel, designated M1 and M2, thereby establishing experimental replicates. As illustrated in Figure 2, representing the evolution of permeability over the filtration time, the experiments were conducted at imposed constant permeate fluxes of 90 and 120 L h^−1^ m^−2^. Membrane permeability was calculated continuously from the transmembrane pressure during filtration. The initial filtration stage, prior to the commencement of backwashing, constitutes a critical phase in the filtration process. During this stage, a pronounced decline in membrane permeability was observed, with the LiqTech membrane (A) exhibiting a reduction of approximately 300 L h^−1^ m^−2^ bar^−1^, whereas the membrane from the other manufacturer (B) showed a decrease of about 50 L h^−1^ m^−2^ bar^−1^. The first backwashing step led to partial removal of fouling, restoring the permeability to 900 L h^−1^ m^−2^ bar^−1^ for the LiqTech membrane (A) and 300 L h^−1^ m^−2^ bar^−1^ for the other manufacturer’s (B). These results clearly indicate that membranes from manufacturer B were considerably more susceptible to fouling, while the LiqTech membranes demonstrated a markedly higher resistance to fouling.

**Figure 3:**
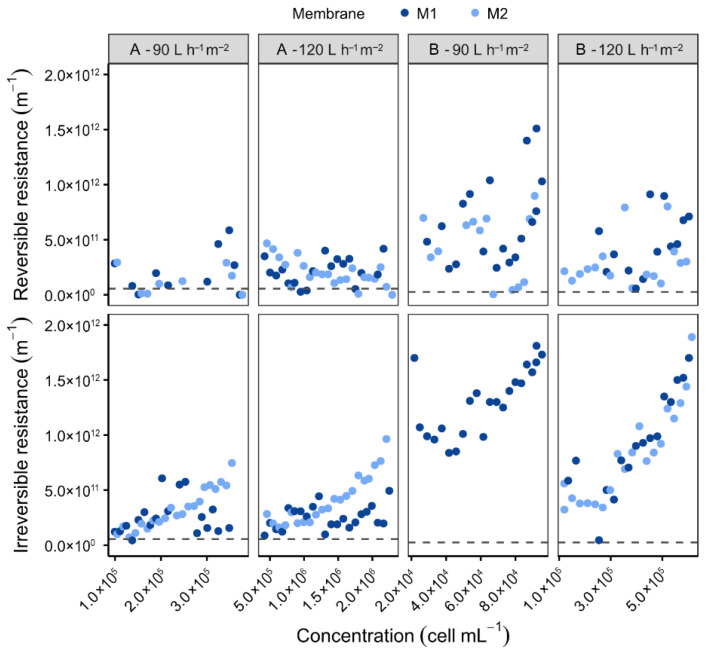
Variation in reversible and irreversible resistances as a function of concentration for two membranes from different manufacturers (A and B) and for two permeate fluxes: 90 and 120 L h^−1^ m^−2^. [Backwash type: BP; BP frequency: 10 min; Recirculation: 3500 L h^−1^; Microalgae: *O. aurita;* Dashed line = R_m_].

Over the course of these experiments, the LiqTech membranes (A) exhibited superior resistance to fouling (Figure 3). The irreversible resistance values of the LiqTech membranes were approximately half those measured for membranes from the other manufacturer (B), ranging from 6.42 × 10^10^ to 9.64 × 10^11^ m^−1^ compared to 2.01 × 10^12^ m^−1^ from manufacturer B. Under identical cell concentrations (5 × 10^5^ cells mL^−1^) and a permeate flux of 120 L h^−1^ m^−2^, the irreversible resistance of the membranes from manufacturer B reached approximately 1 × 10^12^ m^−1^, whereas that of membrane A remained substantially lower.

**Figure 4:**
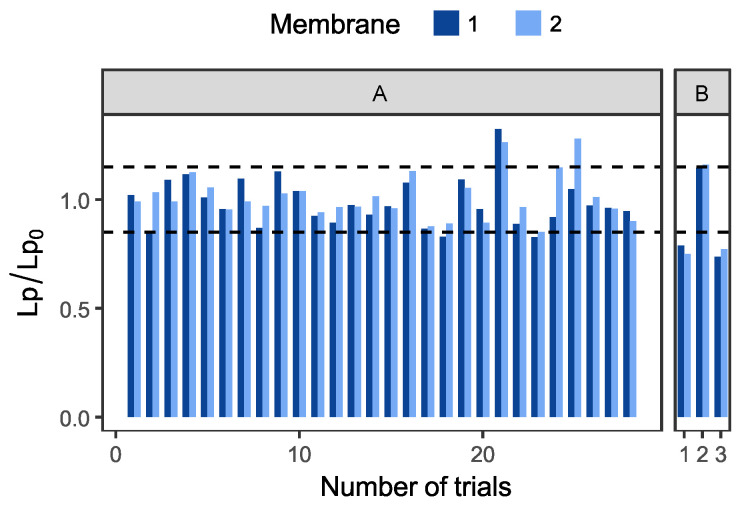
Permeability ratio relative to initial membrane permeability over filtration step [A: LiqTech Membrane; B: Other manufacturer; Dashed lines = 15% interval].

The final parameter studied for the purpose of determining the membranes to be used was regeneration efficiency. In order to facilitate industrial-scale implementation, both filtration and membrane cleaning parameters were systematically refined to ensure effective recovery of permeability following each cycle. The validation of cleaning efficiency was determined by the maintenance of permeability recovery within a margin of ±10% of the reference value. This acceptable margin is attributed to the high permeability and measurement variability of silicon carbide membranes. The final cleaning protocol developed combined backwashing for cell recovery with sequential alkaline, oxidative, and acid steps. The application of 100 ppm sodium hypochlorite, following an overnight soak, was introduced between base and acid washes with the objective of enhancing the removal of organic residues. For outdoor cultures, soda washing was imperative due to the mixed organic load, as chlorine alone (even at 500 ppm, as reported in the literature [45,46]) was inadequate and potentially damaging to organic membranes. This concern is not pertinent in this context, given the high resistance of silicon carbide to oxidation. Acid washing was incorporated into the process with the objective of eliminating mineral deposits from the metal-rich borehole water. The resulting protocol is low-cost, readily automated, and compatible with non-continuous harvesting cycles. However, the effectiveness of the protocol in restoring the initial permeability of the membranes exhibited variation depending on the manufacturer. As demonstrated in Figure 4, the membrane regeneration remained stable (Lp/Lp_0_ = 100 ± 15%) over a period of more than 60 cycles for the LiqTech (A). However, it was less effective on membranes from the other manufacturer (B) that exhibited reduced effectiveness, requiring two successive cleaning cycles. These results confirmed the presence of significant fouling on the membranes produced by the manufacturer (B) and the continuation of testing with the LiqTech (A) membranes was deemed to be a worthwhile endeavour. The enhanced fouling resistance of the LiqTech (A) led to elevated permeate fluxes, a more effective response to backwashing, and a more efficient cleaning process, thereby reducing the overall cleaning time by half. It can be hypothesised that these discrepancies are associated with variations in pore size, with larger pores potentially favouring pore blocking and consequently restricting overall performance [43,45].

### 3.2. Optimisation of Filtration Parameters

The filtration parameters (permeate flux rate, concentration factor and backwashing) were optimised, as were the final concentration of the suspension and the integrity of the microalgae. Due to the seasonal nature of microalgae production, it was not possible to undertake a comprehensive evaluation of all filtration parameters for each species. However, the findings obtained are sufficient for establishing filtration conditions and determining the value of membrane processes for this stage of production.

#### 3.2.1. Influence of Permeate Flux

**Figure 5:**
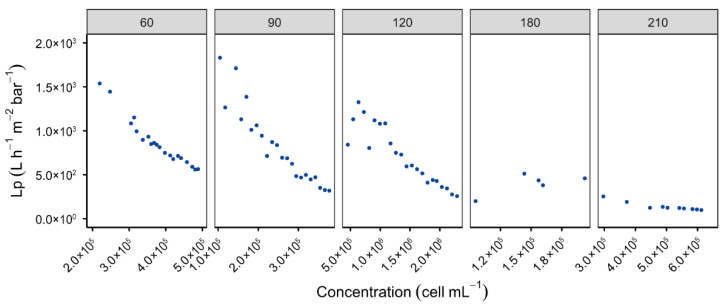
Variation in the permeability of M1 membrane at 20 °C as a function of cell concentration in the retentate for different permeate fluxes (L h^−1^ m^−2^) imposed [Backwash type: BP; BP frequency: 10 min; Membranes: LiqTech; Microalgae: *O. aurita*].

To align with industrial objectives, the process of filtration was performed under constant permeate flux with recirculation. This approach was adopted in order to avoid pressure fluctuations, which are typical of open-loop systems, and to limit mechanical stress on *O. aurita* cells [45]. This configuration also facilitates fouling control and energy cost estimation. The study involved the testing of permeate fluxes ranging from 60 to 210 L h^−1^ m^−2^, with analysis focusing on data immediately before and after backwashing in order to facilitate the calculation of resistance. As demonstrated in Figure 5, an enhancement in permeate flux was observed to result in a concomitant reduction in permeability. The highest levels of performance were observed within the range of 60 and 120 L h^−1^ m^−2^, with permeability decreasing from approximately ~1300 to 300 L h^−1^ m^−2^ bar^−1^ as cell concentration increased from 5 × 10^5^ to 2.5 × 10^6^ cells mL^−1^. At higher fluxes (180–210 L h^−1^ m^−2^), fouling intensified rapidly, reducing permeability to ~100 L h^−1^ m^−2^ bar^−1^, with backwashing proving ineffective.

**Figure 6:**
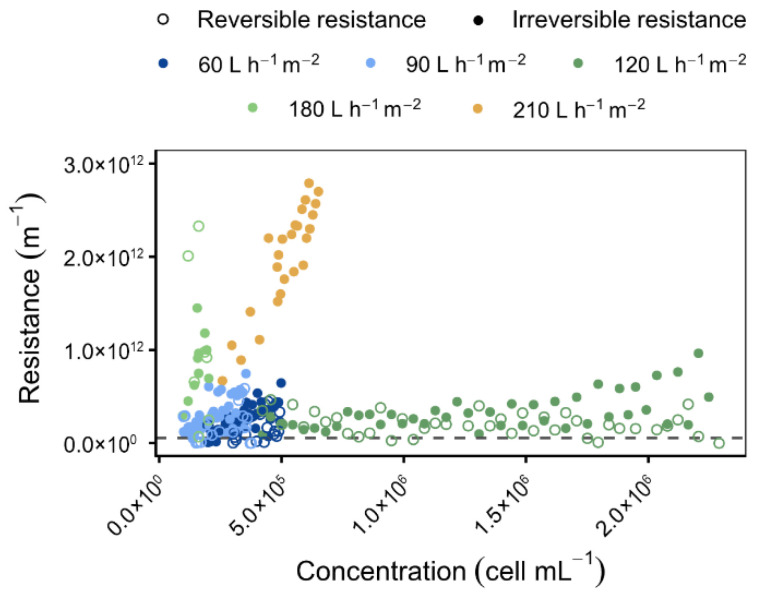
Variation in the fouling resistance as a function of cell concentration in the retentate for different permeate fluxes imposed [T = 20 °C, Backwash type: BP; BP frequency: 10 min; Membranes: LiqTech; Microalgae: *O. aurita*; Dashed line = R_m_].

As illustrated in Figure 6, the corresponding resistances are dominated by irreversible resistance at 180–210 L h^−1^ m^−2^, independent of cell concentration, thereby confirming irreversible fouling under these conditions. At lower fluxes (60–90 L h^−1^ m^−2^), both resistances exhibited a more pronounced increase in response to concentration, a phenomenon that is likely attributable to the presence of non-algal compounds within the culture. Monitoring resistance as a function of concentration thus enables process optimisation and early detection of culture quality issues. The optimal harvesting range for *O. aurita* was identified as between 60 and 120 L h^−1^ m^−2^, ensuring high permeability and controlled fouling.

#### 3.2.2. Culture Concentration Factor

**Figure 7:**
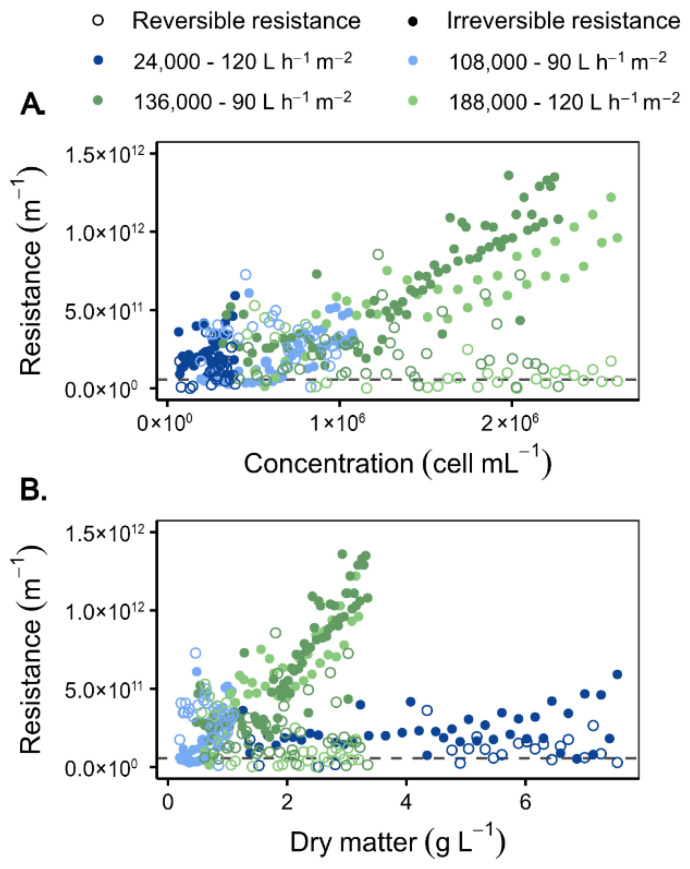
Variation in reversible and irreversible resistances as a function of (**A**) concentration in the retentate or (**B**) dry matter for different initial concentrations and for two permeate fluxes: 90 and 120 L h^−1^ m^−2^. [Backwash type: BP; BP frequency: 10 min; Recirculation: 3500 L h^−1^; Membranes: LiqTech; Microalgae: *O. aurita*; Dashed line = R_m_].

The influence of culture characteristics on membrane fouling was further examined by analysing permeability and resistance variations as a function of cell and dry matter concentrations. Figure 7 presents the evolution of permeabilities and fouling resistances as a function of cell concentration (Figure 7A) and dry matter concentration (Figure 7B) for cultures with different initial cell concentrations. Contamination sources (e.g., algae, bacteria, predators) were not characterised individually; suspensions are thus considered as mixtures of *O. aurita* cells and particles > 1 µm. Filtration tests were performed at permeate fluxes of 90 and 120 L h^−1^ m^−2^ over a wide range of cell concentrations (24 × 10^3^–188 × 10^3^ cells mL^−1^). The magnitude of the reversible resistance remained constant (~5 × 10^11^ m^−1^) beyond 6 g L^−1^ of dry matter, irrespective of the operating conditions. Irreversible resistance exhibited a substantial increase above 100 × 10^3^ cells mL^−1^, reaching 1.5 × 10^12^ m^−1^, accompanied by a modest decline in permeability. At low cell densities (≈25 × 10^3^ cells mL^−1^), the primary cause of fouling was suspended matter, resulting in an irreversible resistance of ~1 × 10^11^ m^−1^. Such low concentrations are indicative of suboptimal, likely contaminated cultures, thereby explaining the higher degree of fouling observed despite the lower cell content. These findings are in accordance with the conclusions of Bamba et al. (2021) [34], who demonstrated that an increase in feed concentration leads to an enhancement in irreversible fouling, resulting in a shift from cake formation (≤0.25 g L^−1^) to adsorption and pore blockage (≥0.5 g L^−1^). Despite the limitations of a direct comparison due to the variable correlation between dry matter and cell concentration in *O. aurita*, the observed threshold of ~100 × 10^3^ cells mL^−1^ or approximately 0.39 g L^−1^ is consistent with the values reported by Bamba et al. (2021) [34].

The findings underscore the significance of presenting data in terms of both dry matter concentration and cell density, as each parameter offers complementary insights. The dry matter index has been shown to reflect the total particulate load, which in turn influences membrane fouling. In contrast, cell density has been demonstrated to relate directly to biomass recovery efficiency. However, the phenomenon of membrane fouling appears to be predominantly influenced by the cell concentration. Given the industrial scale of the study, this approach is more relevant, and therefore, the results will be presented according to cell concentration. The observations are consistent with the known effects of matrix composition on membrane performance in seawater treatment, where organic or inorganic compounds influence fouling mechanisms [47,48,49]. Even at cell densities above 2.5 × 10^6^ mL^−1^, stable fluxes of 90–120 L h^−1^ m^−2^ were maintained. In comparison to standard harvest concentrations (8–10 × 10^4^ cells mL^−1^), a concentration factor of 25 was achieved without optimisation, primarily due to the system’s dead volume, providing performance comparable to decantation but without cell loss in the permeate (see Section 3.4).

#### 3.2.3. Backwash Parameters

**Figure 8:**
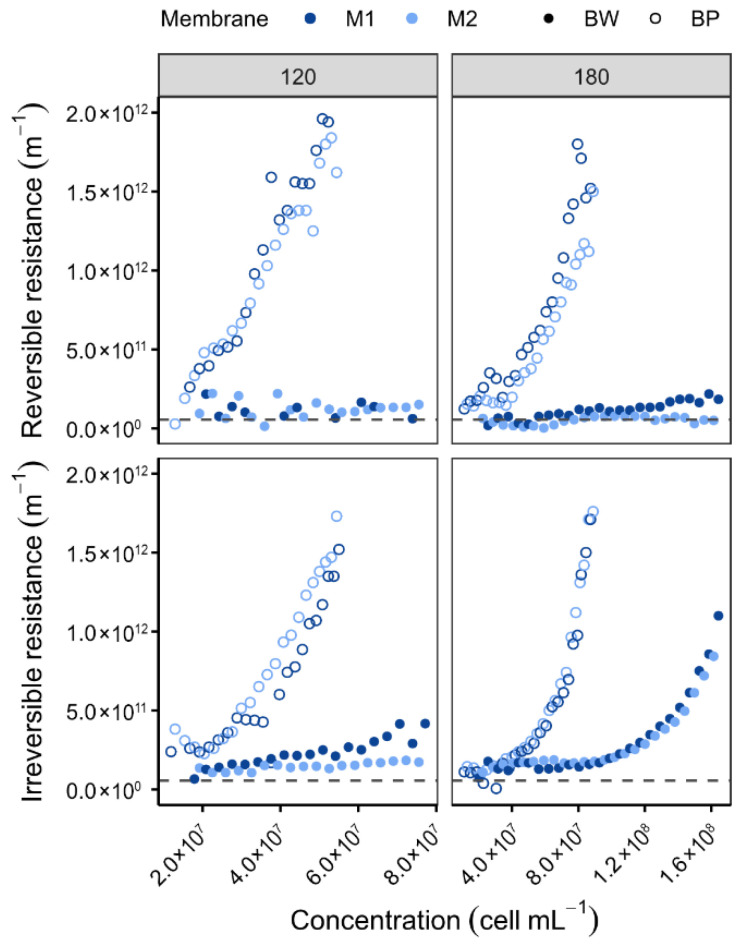
Variation in resistance at 20 °C of membranes as a function of retentate concentration for different permeate fluxes and backwash types [Backwash frequency: 10 min; Backwash duration: 5 s; Membranes: LiqTech; Microalgae: *P. tricornutum*; Dashed line = R_m_].

The impact of backwash (BW) and backpulse (BP) cleaning methods was evaluated during *P. tricornutum* filtration, a species with high initial cell concentrations (>2.5 × 10^6^ cells mL^−1^). Tests were conducted at permeate fluxes of 120 and 180 L h^−1^ m^−2^ (Figure 8) to enhance fouling contrast. The findings indicate that BW exhibits superior efficacy in the mitigation of membrane fouling when compared with BP. The efficacy of the process is characterised by the maintenance of reversible and irreversible resistances below 5 × 10^11^ m^−1^, even at cell concentrations as high as 1.2 × 10^8^ cells mL^−1^. It has been observed that under conditions of BP, both reversible and irreversible resistances increased rapidly and synchronously until reaching 1.5 × 10^11^ m^−1^ at 5 × 10^7^ and 8 × 10^7^ cells mL^−1^ for fluxes of 120 and 180 L h^−1^ m^−2^, respectively. The degree of fouling exhibited was found to be more influenced by the injected water volume, which was 1.5 times higher during BW, than by the impulse strength. These findings contrast with those reported by Bhave et al. (2012) [50], who observed higher BP efficiency for *Nannochloropsis* sp., a smaller, non-siliceous alga. The observed discrepancy is presumably attributable to species-specific morphology, cell size, and extracellular polymeric substances (EPS) production.

**Figure 9:**
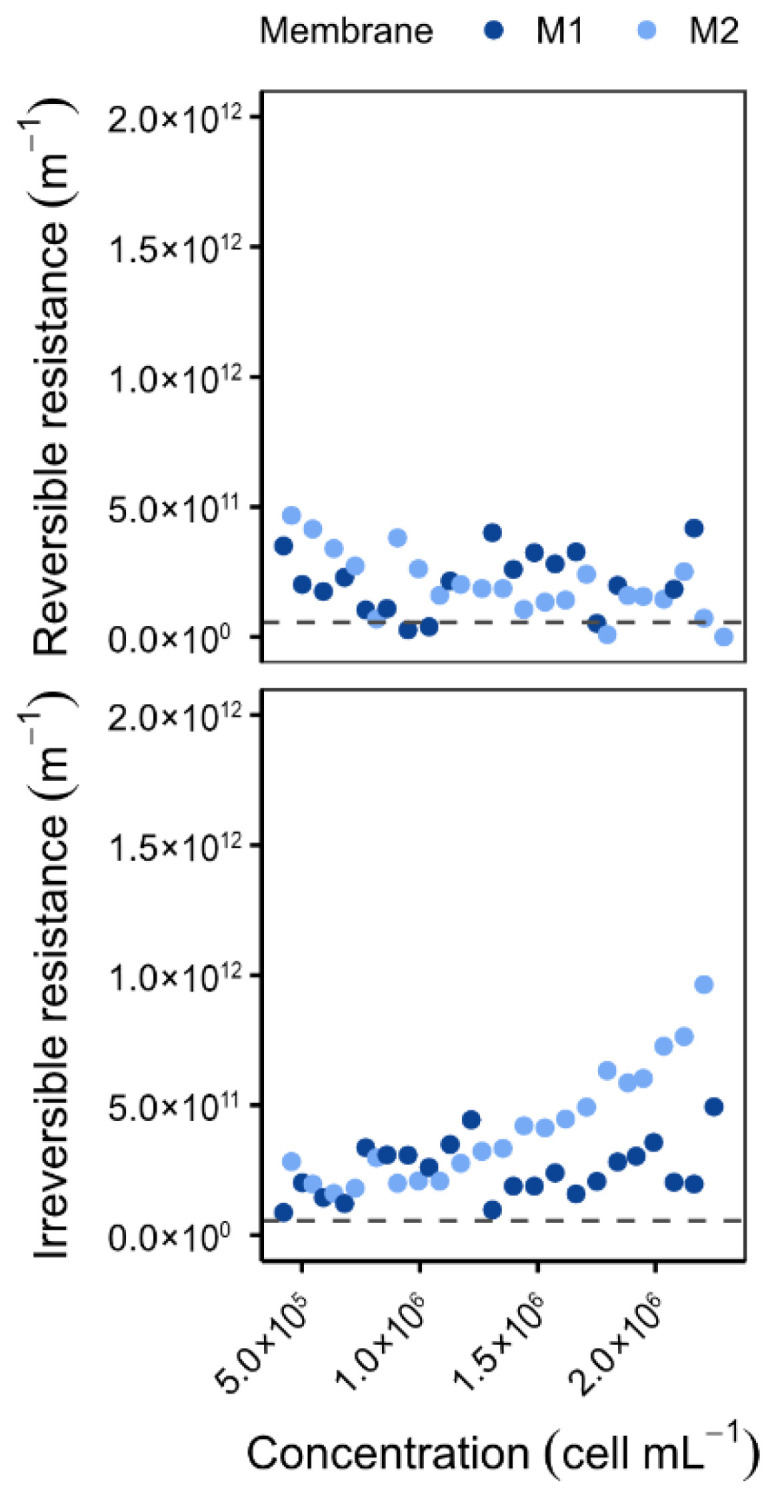
Variation in reversible and irreversible fouling resistance at 20 °C as a function of retentate concentration [Backwash type: BP; Flux: 90 L h^−1^ m^−2^; Membranes: LiqTech; Microalgae: *O. aurita*; Dashed line = R_m_].

In a similar manner, backwashing was considered in the context of *O. aurita* (Figure 9). *O. aurita* is the largest microalga studied in this work. In view of its dimensions, which can attain a magnitude of 100 µm, the formation of a porous cake that is comparatively effortless to remove was anticipated. However, *O. aurita* is frequently contaminated and produces more extracellular polymers (proteins, polysaccharides, humic acids, lipids) [51], a process that is intensified by the presence of predators [52], forming dense cakes that require BP cleaning [24]. Consequently, even at low concentrations of approximately 2 × 10^6^ cells mL^−1^ (i.e., in comparison to Figure 8), irreversible resistance appears to be elevated. The results of the present study demonstrate the pivotal function of backwash parameters (type, frequency, and duration) in optimising filtration performance and scalability.

### 3.3. Cellular Integrity: Focus on Dunaliella salina

**Figure 10:**
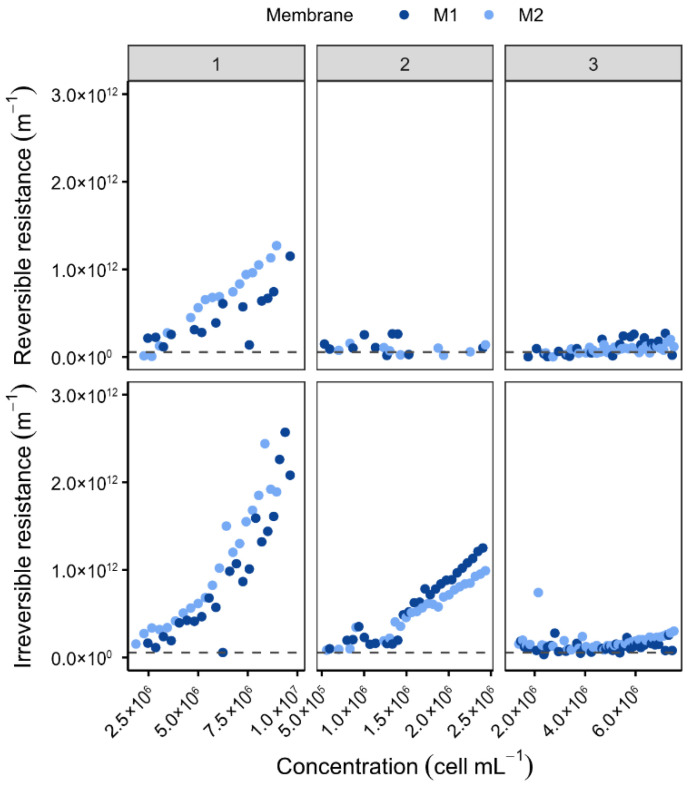
Variation in membrane resistance at 20 °C as a function of retentate concentration for three tests, variation in flux, type of backwash and circulation speed [1. BW/120 L h^−1^ m^−2^/3500 L h^−1^; 2. BP/90 L h^−1^ m^−2^/2000 L h^−1^; 3. BW/90 L h^−1^ m^−2^/2000 L h^−1^. Backwash frequency: 10 min; Membranes: LiqTech; Microalgae: *D. salina*; Dashed line = R_m_].

It is imperative that optimisation of filtration parameters is undertaken with due consideration for microalgal cell integrity. No cell lysis was observed for *P. tricornutum* and *O. aurita*, two species of diatoms with highly resistant siliceous frustules; therefore, cell integrity tests focused on *D. salina*, a species without a cell wall and therefore more sensitive to shear stress. Three trials were performed (Figure 10). In the first experiment, under conditions applied to the other species (120 L h^−1^ m^−2^; 10 min BW; 3500 L h^−1^ circulation, Re = 6380), severe fouling occurred and cell recovery was limited to 64%, despite no cells appearing in the permeate, indicating lysis during filtration. The accelerated rise in irreversible resistance has been hypothesised to be associated with the degradation of algal cells and the subsequent release of cellular compounds, resulting in the fouling of the membrane in the porous media. The estimated wall shear stress, as determined by CFD simulation, based on [53] and references therein, (≈18 Pa) exceeded the 4.2 Pa rupture threshold reported by Monte et al. (2018) [45].

A second experiment was conducted, in which the reduced flux (90 L h^−1^ m^−2^) and shear (2000 L h^−1^ circulation; Re = 3700; τ ≈ 8 Pa) were reduced, with BW being substituted for BP. The initial shear rate of 17,140 s^−1^ was reduced to 6900 s^−1^; however, the transition from BW to BP changes the ΔP/ΔT from 0.29 bar s^−1^ to ~3 bar s^−1^. While there was an initial improvement in performance, irreversible resistance increased rapidly beyond 1 × 10^6^ cells mL^−1^, and cell recovery dropped to 31%. This confirmed increased cell damage. A third configuration was found to maintain these hydraulic conditions whilst reinstating BW, thus resulting in optimal performance. This was evidenced by a 94% viable cell recovery rate (in two trials), which in turn resulted in reversible and irreversible resistances that were found to be close to intrinsic membrane values. The data demonstrate that high shear and BP impulses promote cell rupture. The mitigation of shear stress was achieved through the implementation of centrifugal pumps, a recirculation loop, and inlet water boxes, which served to limit the effect of compression and decompression. The moderate shear applied (≈8 Pa) effectively balanced the processes of fouling control and cell preservation. The filtration process was subjected to rigorous testing at two distinct salinities (35 and 110 g L^−1^), which are representative of the environmental conditions that *D. salina* typically experiences in its natural habitat [54]. Comparable permeabilities and resistances were obtained at both salinities, with maintained cell integrity even at high concentrations (~4 × 10^5^ cells mL^−1^), whatever the salinity.

**Figure 11:**
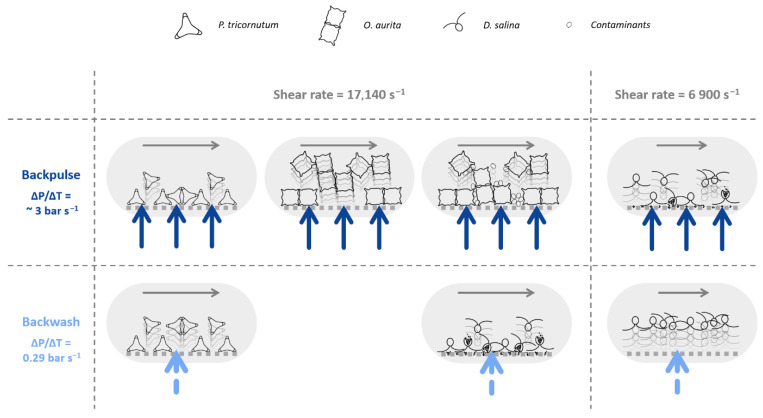
Schematic representation of membrane unfouling according to backwash type, shear rate, and the microalgae studied.

Finally, the behaviour of the three microalgae during filtration can be represented schematically in Figure 11. This schematic demonstrates the enhanced efficiency of backwashes in removing *P. tricornutum* cells that have accumulated on the membrane surface in comparison with backpulses. Furthermore, the study provides a compelling illustration of the influence of culture contamination in *O. aurita*, demonstrating that the presence of contaminants results in the formation of a more compact filtration cake, thereby reducing the effectiveness of backpulses. Finally, it demonstrates the degradation of *D. salina* cells during tests conducted at excessively high shear rates or under backpulse conditions, as well as the effectiveness of combining backwash with low shear rate in both removing fouling and preserving algal cell integrity.

### 3.4. Harvesting Efficiency

The harvesting procedures employed by Innovalg vary according to the species in question. *O. aurita* is pre-concentrated via rotating drum filtration, followed by spontaneous sedimentation, while *P. tricornutum* undergoes alkaline flocculation (NaOH) and successive settling before neutralisation with HCl (pH 7.5). The industrial flocculation of *D. salina* has not yet been achieved. The industrial-scale volume concentration factors (VCF) obtained by flocculation/decantation were 55 for *O. aurita* and 40 for *P. tricornutum* (Table 2). The processing volumes were 55 and 35 m^3^ per batch, respectively. On a semi-industrial scale, membrane filtration yielded VCFs of 19.0 for *O. aurita*, 20.8 for *D. salina*, and approximately 40 for *P. tricornutum*. The comparatively lower values are attributable to the 10 L dead volume of the pilot unit and limited daily operation (10 h). The mean cell recoveries in industrial harvesting were 55.5 ± 14.7% (*O. aurita*) and 68.6 ± 3.1% (*P. tricornutum*), whereas optimised filtration achieved 88%, 94%, and 91 ± 11%, respectively (Table 2). No microalgae were detected in the permeate, indicating near-complete retention. Unrecovered cells correspond to surface deposition on the membrane. The findings of this study demonstrate the merits of membrane filtration in terms of both recovery yield and biomass integrity, with the notable advantage of not requiring chemical additives. Furthermore, membrane filtration has been shown to markedly reduce the number of preconcentration steps, equipment, and processing time.

The extent of reported VCF ranges within the existing literature varies considerably. This has been found to span a range from 5 to 154, contingent on both species and operating conditions. The range of values for *Chlorella* sp. is from 5 to 40 [55], for *Nannochloropsis* sp. from 75 [50], and for *Tetraselmis suecica* from 100 [5]. Utilising LiqTech SiC membranes (0.04–1 µm), Safafar (2017) [35] obtained VCFs of 20–70 for *Chlorella sorokiniana* and 5–35 for *D. salina*, *C. vulgaris* and *P. tricornutum*, which is consistent with the present findings. The energy consumption for membrane preconcentration averaged at 1.2 kWh m^−3^, which is significantly lower than Innovalg’s current pre-harvesting process (2.2 kWh m^−3^). For comparison, the literature values range from 0.35 kWh m^−3^ (*D. salina*) [42], 0.49–0.57 kWh m^−3^ for *N. gaditana* and *C. sorokiniana* using organic ultrafiltration membranes (30 nm) [56], 0.7 kWh m^−3^ for *Nannochloropsis* sp. [50], and up to 2.23 kWh m^−3^ for *Scenedesmus* sp. [57]. The final centrifugation stage adds ~1.96 kWh m^−3^ irrespective of the preconcentration method. These results provide validation of the energy efficiency and industrial potential of SiC membrane filtration for microalgal biomass harvesting. From an industrial perspective, the volume of biomass to be processed was estimated at 45 m^3^ per harvesting event, with an allocated filtration time of 12 h, assuming automated operation during the night. At the lowest permeate flux level that was tested (90 L h^−1^ m^−2^), a total membrane area of approximately 42 m^2^ would be required, corresponding to 127 ceramic membranes (0.33 m^2^ each), arranged in two parallel K67 housings. According to the latest industrial references, the estimated investment cost of a unit equipped with two K67 housings is approximately €200,000. Ceramic membranes characteristically demonstrate an operational lifespan of approximately 10 years, while auxiliary equipment such as pumps and sensors typically necessitates replacement after approximately 5 years. It should be noted that additional costs may arise in the event of the integration of advanced automation, optimised backwashing strategies, or thermal control systems. OPEX values will be adjusted after testing on this industrial unit based on the microalgae produced. The order of magnitude for energy consumption will be adjusted to around 2.2 kWh m^−3^.

In order to reduce this cost and as a possible solution to the problem, the introduction of Dean’s vortices is suggested. Dean vortices emerge in curved or helical channels as a consequence of the interplay between centrifugal forces and fluid viscosity [58]. Secondary flows have been shown to enhance mixing, disrupt boundary layers, and reduce membrane fouling by limiting particle deposition, see [59,60] and references therein. Due to the propensity of solids to accumulate at the vortex centre, the overall effect is one of enhanced permeability and membrane durability. As demonstrated in previous studies, the induction of Dean vortices in spiral or curved membrane modules has been shown to significantly enhance performance, with reported increases ranging from 50 to 70% in flux rate and 40–90% in recovery in comparison to linear channels [61,62,63]. Recent studies have investigated the scaling up of such designs, though their application to industrial ceramic membranes remains limited. The newly developed ceramic membranes developed by TAMI Industries, featuring helical channels, were investigated in this study, and it was demonstrated that vortex generation is advantageous in this context: permeate fluxes increased by up to 1.6× for *D. salina* and 46% for *P. tricornutum*, along with over 30% higher energy efficiency. Preliminary findings also indicate enhanced algal recovery, which is presumably associated with cell aggregation within vortices.

## 4. Conclusions

The present study focused on the development of semi-industrial mineral microfiltration for the purpose of microalgae harvesting. The concentration of algal biomass on a large scale is a complex process due to the biodiversity of species and their morphological and rheological traits, which have a significant impact on membrane fouling. The three microalgae species examined demonstrated divergent fouling behaviours. Mineral membranes, despite their higher cost, are deemed suitable due to their high permeability, backwash capability, ease of cleaning, and chemical and thermal resistance [34]. Silicon carbide membranes, characterised by low tortuosity, facilitate the efficient harvesting of the three microalgae species targeted in this study. The performance of the filtration process was found to be contingent upon the characteristics of the algal suspension. Optimal permeate fluxes ranged between 90 and 120 L h^−1^ m^−2^, reaching 180 L h^−1^ m^−2^ for *P. tricornutum*, a low-viscosity species with limited fouling [35,64]. The cleaning strategy exhibited a species-specific variance. In the case of *O. aurita*, the backpulse approach proved most efficacious, attributable to the dense biofilm characteristic of this species. Conversely, backwashing exhibited superiority in preserving flux for *P. tricornutum*. For *D. salina*, which lacks a siliceous wall and is sensitive to shear, gentler conditions (90 L h^−1^ m^−2^ and reduced tangential velocity) were applied to prevent cell rupture; backwashing was preferred over backpulse. Membrane filtration was compared with flocculation and decantation. Notwithstanding the inherent limitations in terms of scale, comparable volume concentration factors were attained, notably in the case of *P. tricornutum*. Membrane filtration has been shown to provide nearly complete cell recovery, with efficiencies ranging from 40% to 70% when compared to conventional methods. Furthermore, this approach has been demonstrated to preserve biomass quality, thereby avoiding the chemical degradation that can be caused by flocculants [65]. Preliminary tests with Dean vortex-generating membranes have demonstrated a reduction in energy consumption during the concentration phase. The present study is part of a larger research programme, the details of which will be published at a later date.

## Figures and Tables

**Table 1:** Membrane characteristics.

Manufacturer.	LiqTech—Manufacturer A	Manufacturer B
Material	SiC	SiC
Geometry	Multi-channel tubular	Multi-channel tubular
Filtration direction	Internal-external	Internal-external
Pore size (µm)	0.20	0.25
Permeability at 20°C (L h^−1^ m^−2^ bar^−1^)	7000	13,500
Surface filtering (m^2^)	0.33	0.33
Number of Channels	30	31
Internal Diameter (mm)	3	3
pH resistance	0–14	0–14

**Table 2:** Concentrated volume, average VCF and cell recovery rate according to the harvesting technique.

	Flocculation/Settling		Membrane Filtration	
	Volume (m^3^)	VCF	Volume (L)	VCF
*O. aurita*	53.8	55	185.8	19
*P. tricornutum*	34.2	40	333.0	39
*D. salina*	-	-	186.5	21
	C_0_ (kcell mL^−1^)	Cells preserved (%)	C_0_ (kcell mL^−1^)	Cells preserved (%)
*O. aurita*	51.4	55.5 ± 15%	93.5	88 ± 10%
*P. tricornutum*	5050	68.6	3203	91 ± 11%
*D. salina*	-	-	454	94 ± 11%

## Data Availability

The raw data supporting the conclusions of this article will be made available by the authors on request.
